# Alternative Splicing, Epigenetic Modifications and Cancer: A Dangerous Triangle, or a Hopeful One?

**DOI:** 10.3390/cancers14030560

**Published:** 2022-01-22

**Authors:** Francisco Gimeno-Valiente, Gerardo López-Rodas, Josefa Castillo, Luis Franco

**Affiliations:** 1 Department of Oncology, Institute of Health Research, INCLIVA, 46010 Valencia, Spain; fgimenovaliente@gmail.com (F.G.-V.); gerardo.lopez@uv.es (G.L.-R.); pepa.castillo@uv.es (J.C.); 2 Department of Biochemistry and Molecular Biology, Universitat de València, 46010 Valencia, Spain; 3 Centro de Investigación Biomédica en Red en Cáncer (CIBERONC), 28029 Madrid, Spain

**Keywords:** epigenetics, alternative splicing, chromatin, cancer, histone acetylation, histone methylation, DNA methylation

## Abstract

**Simple Summary:**

Epigenetics studies the alteration of gene expression without changing DNA sequence and very often, epigenetic dysregulation causes cancer. Alternative splicing is a mechanism that results in the production of several mRNA isoforms from a single gene and aberrant splicing is also a frequent cause of cancer. The present review is built on the interrelations of epigenetics and alternative splicing. In an intuitive way, we say that epigenetic modifications and alternative splicing are at two vertices of a triangle, the third vertex being occupied by cancer. Interconnection between alternative splicing and epigenetic modifications occurs backward and forward and the mechanisms involved are widely reviewed. These connections also provide novel diagnostic or prognostic tools, which are listed. Finally, as epigenetic alterations are reversible and aberrant alternative splicing may be corrected, the therapeutic possibilities to break the triangle are discussed.

**Abstract:**

The alteration of epigenetic modifications often causes cancer onset and development. In a similar way, aberrant alternative splicing may result in oncogenic products. These issues have often been individually reviewed, but there is a growing body of evidence for the interconnection of both causes of cancer. Actually, aberrant splicing may result from abnormal epigenetic signalization and epigenetic factors may be altered by alternative splicing. In this way, the interrelation between epigenetic marks and alternative splicing form the base of a triangle, while cancer may be placed at the vertex. The present review centers on the interconnections at the triangle base, i.e., between alternative splicing and epigenetic modifications, which may result in neoplastic transformations. The effects of different epigenetic factors, including DNA and histone modifications, the binding of non-coding RNAs and the alterations of chromatin organization on alternative splicing resulting in cancer are first considered. Other less-frequently considered questions, such as the epigenetic regulation of the splicing machinery, the aberrant splicing of epigenetic writers, readers and erasers, etc., are next reviewed in their connection with cancer. The knowledge of the above-mentioned relationships has allowed increasing the collection of biomarkers potentially useful as cancer diagnostic and/or prognostic tools. Finally, taking into account on one hand that epigenetic changes are reversible, and some epigenetic drugs already exist and, on the other hand, that drugs intended for reversing aberrations in alternative splicing, therapeutic possibilities for breaking the mentioned cancer-related triangle are discussed.

## 1. Introduction: Defining the Triangle

In spite of the advances in precision medicine [1], cancer is still one of the most feared and deadly diseases in the world. Its incidence is steadily raising [2], especially at older age, and this, apart from the concomitant human suffering and loss of lives, will considerably increase the economic and social burden of the disease [3]. The vital role of the genome in cancer development has long been considered, especially during the last decade; after the seminal paper by Stratton et al. [4], a considerable effort has been made to identify genes harboring driver mutations. While this search has reported a significant advance in our understanding of the molecular causes of cancer, in a certain sense it has eclipsed a fundamental fact, namely that the factors controlling gene expression are essential players in the game of gene functionality. Alterations in epigenetic factors may regulate gene expression in a way that emulates the effects of genetic mutations, leading to what has been aptly called epimutations [5]. It is, therefore, no wonder that the study of cancer-related epigenetic alterations has received much attention in the recent years. The crosstalk between genetic and epigenetic aberrations as the driving force of malignant transformation is a matter of debate and the predominance of one or other cause hangs in the balance. As concluded by Nebbioso et al. [6], a better knowledge of genome–epigenome interplay will be crucial to answer this question.

Epigenetic modifications cause changes in gene expression without alterations in the DNA sequence, resulting in the acquisition of distinct phenotypic traits, often related to oncogenicity [5,7]. Epigenetic factors include covalent modifications of DNA and histones and the inclusion of histone variants in nucleosomes. Remodeling of chromatin and the binding of non-coding RNAs (ncRNAs) may also be considered as epigenetic factors.

DNA and histone modifications are reversible, enzyme-driven processes. The enzymes that catalyze the introduction of epigenetic marks are generally named “writers”, while “erasers” is the name given to the enzymes that remove the marks. For an epigenetic mark to fulfil its regulatory function a “reader” is required, in the same manner that a barcode is meaningful only when a scanner capable of reading it is available. The readers are protein molecules carrying one or several domains, which specifically interact with a combination of epigenetic marks or with a single one.

Although there are other modifications in DNA, the most relevant ones are the methylation and the hydroxymethylation of the cytosine ring at its C5 atom in CpG sequences. The methyl group is transferred from S-adenosylmethionine and the fundamentals of DNA methylation have been recently reviewed with a special mention to cancer [8]. The writers, readers and erasers of DNA methylation are summarized in Table S1.

The nature and roles of histone post-translational modifications (PTMs) are more numerous. The writers, readers and erasers of the most relevant ones, acetylation and methylation, are summarized in Tables S2 and S3, but other minor and yet functionally interesting modifications have been described (reviewed in [9]). Histone acetylation is ordinarily related to transcriptional activation, while methylation may result in activation or repression depending upon the nature of the modified histone and residue. Importantly, after the proposals of Loidl et al. [10] and Allis et al. [11], it is accepted that histone PTMs may play signaling roles.

Within the wide landscape of the functions of DNA and histone modifications, the better documented role is transcriptional regulation and its influence on cancer has been the subject of many reviews (see, for instance, [12,13]). Although histone acetylation contributes to release the higher order of chromatin structure, the persistence of nucleosomes is still an obstacle to transcription. The mechanisms and consequences of chromatin remodeling have been an object of review in recent years (for instance, see [14,15]), as it was the link of this dynamic process with cancer [6,16]. Epigenetic modifications and chromatin remodeling are not independent processes. The remodeling complexes are recruited to selected chromatin loci by specific epigenetic marks due to the presence of reader domains in some of the different components of the complexes [9].

The cause of the frequent occurrence of epigenetic alterations in cancer is, in many cases, a genetic one. The mutation of writers, readers or erasers, as well as of the components of remodeling complexes as was early shown to occur in cancer [17,18,19,20]. Actually, mutations in the enzymes involve histone PTMs which rank among the most frequent ones in cancer [21]. However, it has been suggested that, in some instances, “pure epigenetic” deregulation (i.e., induced by non-genetic stimuli) may cause the classic hallmarks of cancer [22].

A different and yet crucial circumstance has to be considered in the context of gene expression. Most of the mammalian genes possess several exons and usually exhibit alternative splicing (AS), which gives rise to various mature mRNA isoforms. AS is relevant for cancer onset and development (for a recent review, see [23]) and it sometimes occurs that isoforms of a single gene play opposite functions, some of them being oncogenic, while others are not.

AS most often results from exon skipping, but other types, such as alternative 3′ or 5′ splicing sites, mutually exclusive exons and intron retention have been described [24]. The mechanisms involved in AS are very complex and they have been recently reviewed [25]. More than 100 factors, including small nuclear ribonucleoproteins (snRNPs) heterogeneous nuclear ribonucleoproteins (hnRNPs) and enzymes are required to form the intricate complexes termed spliceosomes, which direct the splicing processes. A less-frequent mechanism, back-splicing, gives rise to circular RNA (circRNA) molecules [26] and is gaining attention, because circRNAs play a role in the regulation of tumor development. Other AS-related possibilities exist that lead to multiple transcripts from a single gene. The most common ones are the presence of alternative promoters [27] or transcription termination sites [28].

**Figure 1:**
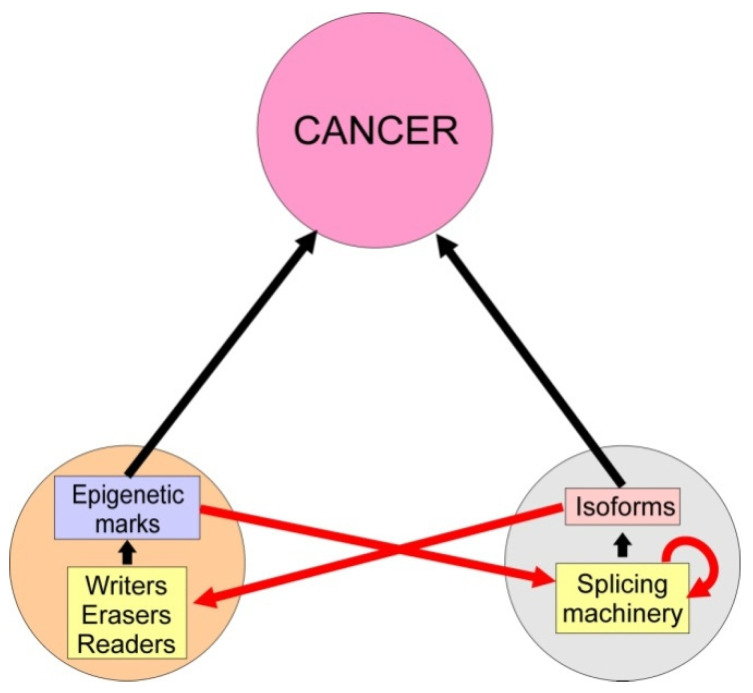
Triangle formed by cancer, epigenetics and alternative splicing. Changes in normal epigenetic marks (epimutations) and aberrant alternative splicing cause cancer in many instances (black arrows). Both causes are mutually interconnected (red arrows), because epigenetic alterations affect the splicing machinery and splicing may alter in several ways the epigenetic modifications. Moreover, alternative splicing may influence the components of the splicing machinery themselves (curved red arrow).

Epigenetic dysregulation or aberrant splicing may be, therefore, fundamental for cancer, but it has to be considered that these two processes are interconnected. On the one hand, splicing occurs co-transcriptionally in about 80% of the genes [29] and both the organization of chromatin and its epigenetic modifications have an influence on the process [30,31]. On the other hand, splicing affects some of the genes coding for writers [32,33,34], erasers [35,36] and readers [37,38,39]. In this way, the triangle formed by cancer, epigenetics and alternative splicing is closed (Figure 1); this is the canvas on which the fabric of the present review is woven. We will not focus on the simple influence of epigenetics or AS on cancer; these issues have been the subject of many review articles in recent years. Rather, we will concentrate on the epigenetic alterations that cause cancer through aberrant AS and in the AS events that result in malignant transformations by altering epigenetic players (Figure 1). The question is further complicated by the fact that some splicing factors can be, in turn, affected by the AS of their pre-mRNAs. This issue will be dealt with in detail in Section 5. The excellent review of Amirkhah et al. [40] covers some of these aspects, but, apart from concentrating in colorectal cancer, they follow a different approach to the problem.

It is obvious that when the inappropriate combination of epigenetic and AS events results in the development of cancer, a harmful situation originates. Nevertheless, providing that epigenetic changes are reversible and that a number of epidrugs are under trial or licensed [41,42], and that drugs exist which are aimed at correcting AS aberrations [43,44,45], the possibility exists of breaking the triangle.

Due to the huge number of data reported in the literature, with some small exceptions, especially in articles of historical interest, only the reports published in the last three/four years and not covered by other published reviews will be mentioned here.

## 2. Epigenetic Modifications That Cause Splicing-Related Alterations Involved in Cancer

The most classical epigenetic modifications, DNA methylation and histone PTMs are first dealt with in the present section in their connection with AS in cancer. The binding of ncRNAs and the remodeling of chromatin, which are often included among epigenetic factors, are next considered. Finally, the influence of epigenetic factors, mainly ncRNAs, on the spliceosome and other components of the splicing machinery splicing is considered.

### 2.1. DNA Methylation

DNA methylation may often occur in CpG islands. When these islands are located in gene promoters, methylation leads to gene silencing and so, this epigenetic modification has been associated with the downregulation of gene expression [46]. CpG methylation occurs too within the gene body, and then is an important factor to select transcriptional isoforms as shown below. Another well-documented effect of DNA methylation on AS is related to the kinetic model, which explains the coupling between transcription and AS. According to this model, a fast elongation speed may result either in the skipping of a given exon or in its inclusion, depending on the strong or weak nature of the splicing sites [47]. This may explain that DNA methylation displays a dual role in AS. For instance, Yearim et al. reported, after a genome-wide analysis, that the splicing of more than 20% of alternative exons is regulated by CpG methylation. In some instances exon skipping is activated, while its inclusion is favored in others [48]. Slow RNA pol II processivity may be caused by the recruitment of the zinc finger CCCTC-binding factor (CTCF). Its binding is inhibited by CpG methylation and this results in the modulation of AS [49]. Moreover, DNA methylation affects nucleosome positioning [50] and dynamics [51], as chromatin structure plays a key role in AS [47].The above considerations serve as a basis for reviewing the recent achievements in the relationships between AS and DNA methylation in cancer. First, there are several cases in which a single gene gives rise to different proteins through a methylation-dependent selection of alternative promoters or transcription start sites (TSSs).

A specific example of alternative TSS usage is provided by the *RASSF1* gene, which gives rise to two alternative protein-coding transcripts, RASSF1A and RASSF1C. The first one is a tumor suppressor, while the other is an oncogene [52]. RASSF1C mRNA is transcribed from a promoter located within an intron of the longer RASSF1A. The promoters for both mRNAs are associated with CpG islands and their methylation level has been recently determined in primary breast cancers as well as in breast cancer cell lines. The RASSF1A CpG island is hypermethylated in all the samples examined, while the island of RASSF1C remains unmethylated. This explain why the oncogenic isoform is expressed in all the cell lines, while the suppressor isoform is only expressed in 2 out of the 10 lines assayed [53].

The *LARP1* gene offers another example of cancer-related DNA methylation-dependent isoform selection. The gene is transcribed to nine curated mRNA isoforms, two of them being translated into proteins. These isoforms have been designated as LI-LARP1 (long isoform) and SI-LARP1 (short isoform). They contain 18 common exons and differ in the first one and in the promoter, which is hypermethylated in SI-LARP1. In consequence, this isoform is silenced and its mRNA is only detected after demethylation [54]. The expressed isoform, LI-LARP1 is involved in ribosome biogenesis and its oncogenic properties have been shown in cervical and non-small cell lung [55], ovarian [56] and colorectal [57] cancers.

In gastric cancer, an imbalance of the ratio of FGFR2-IIIb to FGFR2-IIIc of the gene encoding the fibroblast growth factor receptor 2 has been recently reported. This alteration in AS is concomitant to the promoter demethylation of *FGFR2* and of *ESRP1* [58]. The latter gene codes for the epithelial splicing regulatory protein 1, an RNA-binding protein that regulates AS related to epithelial phenotype. In this context, recent research has revealed an indirect effect of DNA methylation on AS. Transcription of ESRP1, which requires the binding of E2F1, is elevated in many cancer types, but is drastically lowered in cancer cells within hypoxic niches, with the concomitant induction of EMT. CpG methylation at E2F1 target site abolished the binding of the transcriptional factor, but CpG hydroxymethylation does not. Under hypoxic conditions, TET3 activity is reduced and, consequently, CpG hydroxymethylation is substituted by de novo methylation catalyzed by DNMT3A/B and E2F1 is no longer able to bind ESRP1 promoter. Thus, hypoxia leads to the repression of ESRP1, which is followed by the loss of the AS events characteristic of the epithelial phenotype [59].

As mentioned above, intragenic CpG methylation may affect splicing. An in silico genome-wide analysis of DNA methylation revealed a clear correlation between the methylation degree of exons and its inclusion in mature mRNAs. Highly-methylated exons tend to be included, while a low methylation is related to exon skipping [60]. Through an in silico search of the exons in which methylation and inclusion run in parallel, Li et al. [60] found that 260 exons were differentially skipped between normal and cancer lung tissues. Gene ontology analysis allowed them to identify five cancer hallmark processes, the most prominent one being regulation of angiogenesis. This means that DNA methylation-dependent AS is involved in cancer progression. Brother of Regulator of Imprinted Sites (BORIS), a paralogue of CTCF that recognizes the same target sequences, has been shown to bind the PKM gene to direct the alternative splicing of the mutually exclusive exons 9 and 10 [61,62]. The PKM gene encodes the glycolytic enzyme pyruvate kinase and can be transcribed to give two alternative mature mRNAs. PKM1 isoform, containing exon 9, is found in normal tissues, while the exon 10-containing PKM2 isoform, which is translated to an enzyme specific for anaerobic glycolysis, is characteristic of tumor cells. In contrast to its paralogue, BORIS binds both methylated and non-methylated CpG-containing sequences, although it has preference for methylated DNA in some cancer cells [63]. In the case of PKM, chromatin immunoprecipitation (ChIP) experiments showed that BORIS is bound to the highly-methylated exon 10 in a breast cancer cell line, while the methylation level and BORIS binding is low in normal cells. Moreover, inhibition of DNA methylation results in the detachment of BORIS and the shift of PKM2 to PKM1 [61], a result that illustrates the mechanisms that regulate PKM AS in cancer. Interestingly, transcription of CTCFL, the gene coding for BORIS, is regulated by the methylation of its promoter [64]. Further complication results from the influence of ncRNAs on the shift PKM1-PKM2, as will be discussed in Section 2.3.

The AS of CD44, which codes for an adhesion membrane protein, also depends on intragenic methylation [65]. Inactivation of DNMT1 and DNMT3b in HCT116 cells results in the production of CD44s, a short variant of CD44 in which the 9 alternative exons (2–10) have been skipped. This skipping causes an increase in the properties of EMT phenotype and is mediated by the demethylation of intragenic CpGs, a circumstance that, as mentioned [60], is a general fact. The intermediate mechanisms have been recently disclosed. Briefly, methyl CpGs recruit MBD1/2/3, which, in turn, bind the histone methyltransferase SUV39H, the enzyme that catalyzes the introduction of the epigenetic mark H3K9me3 in the surrounding nucleosomes (see Section 2.2). H3K9me3 is recognized by the heterochromatin protein HP1γ and the resulting chromatin structure causes the partial pausing of RNA polII. Probably due to this kinetic effect, the alternative exons are included. On the contrary, when the intragenic CpGs are demethylated, the fast processing of the polymerase leads to the skipping of these exons, with the final result of the acquisition of an EMT phenotype [65]. Therefore, it can be concluded that epigenetic modifications display a complex role in the regulation of AS.

DNA methylation also influences intron retention. Wong et al. [66] reported that methylation around 3′ splice junctions is lower in the flanks of retained than in non-retained introns. This is associated with deficiency in MeCP2 recruitment, which, in turn, may result in a deficient recruitment of splicing factors tethered by MeCP2 [66]. Although these mechanisms occur both in normal and cancerous cells, intron retention has been associated with various oncogenic mechanisms, such as inactivation of tumor suppressors [67]. Apart from the possibility that DNA hydroxymethylation may partially substitute for methylation in tumor cells [66], it has to be noted that intragenic hypomethylation is a common feature in cancers [68]. Of note, the aberrant intron retention in chronic myeloid leukaemia is maintained after remission of the disease, associated with reduced DNA methylation [69]. This finding may add to design novel criteria for estimating remission of cancer.

### 2.2. Histone Modifications

Due to the already mentioned coupling with transcription, histone modifications exert a marked influence on splicing. This effect may be due to either the general influence of histone acetylation on chromatin compaction or to the specific signaling role of histone epigenetic marks. An early example of the first cause is given by the work of Kornblihtt et al., who found that the HDAC inhibitor trichostatin A reduces the inclusion of the fibronectin extra domain I exon [70]. This effect may be explained with the kinetic coupling model [47]. A recent example of this kind is provided by the skipping of exon 4 of the osteopontin gene, *OPN*, which gives rise to the short, highly-oncogenic isoform *OPNc*. This skipping is facilitated by HDAC1 or HDAC2 and by the transcriptional factor RUNX2 [71]. The most obvious explanation is that both RUNX2 and acetylated histones make the RNA polII processivity easier.

**Figure 2:**
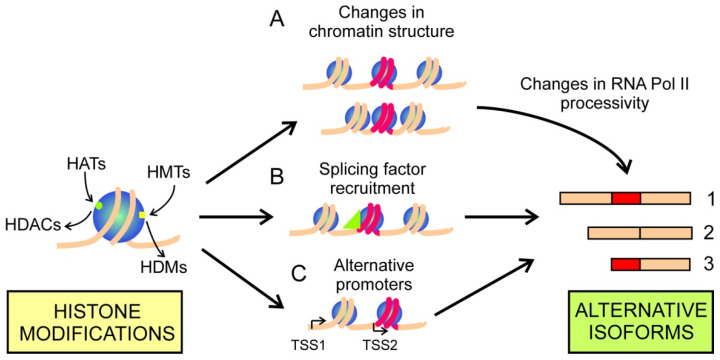
Some examples of the influence of histone modifications in the production of alternative isoforms of mRNA. (**A**) Histone modifications may result in changes of chromatin structure, which modify RNA polII processivity and, due to the kinetic effect, result in inclusion or skipping of an exon (in red), yielding isoforms 1 or 2, respectively. (**B**) Histone modifications may recruit a splicing factor (green triangle), which determines the production of either isoform 1 or 2. (**C**) In this instance, histone modifications determine the selection of alternative promoters, giving rise to either isoform 1 or 3.

The second cause by which histone modifications influence AS is the specific signalling provided by epigenetic marks. Specific marks may be used to recruit factors affecting RNA pol II processivity, thus influencing AS through kinetic coupling. There is still an additional possibility, namely the specific, differential recruitment of splicing factors. Both causes may influence the selection of alternative TSSs and, on these grounds, alternative mRNA isoforms may be transcribed from a single gene (Figure 2). The influence of histone modifications on AS is not completely independent of the effects caused by DNA methylation (see Section 2.1). For instance, H3K4me3 inhibits the DNMTs and, consequently, its presence may induce local changes in CpG islands methylation. The aberrant DNA methylation pattern induced by H3K4me3 in cancers has been recently reviewed [72].

Since the early work of Enroth et al. [73], many efforts have been made to find a “splicing code”, linking specific histone modifications with AS events. By using mouse embryonic tissue developmental data from publicly available databases and a machine learning approach, it has been recently described that there is a link between histone PTMs and AS, and that the interaction of H3K36me3 and H3K4me1 is a strong predictor of splicing in mammalian development [74]. Nevertheless, much work is still needed to obtain a comprehensive set of epigenetic marks that may be used to predict the path linking histone modifications and AS events in each individual gene. Therefore, to determine which of the paths shown in Figure 2 operates in cancer-related processes, one has to rely upon the study of selected cases. The most recent ones are reported in the next paragraphs.

The combined influence of histone modifications and DNA methylation may result in the selection of cryptic promoters. Apart from the cases discussed by Zardo et al. [72], it may be mentioned that of the human *DAPK1* gene. Due to its behavior in colorectal cancer [75] and in clear cell renal carcinoma [76], it is considered as a potential tumor suppressor. It is not expressed in the lung cancer cell line NCI-H1299 because a regulatory CpG island is hypermethylated, but inhibition of DNA methylation restores the expression of the gene [77]. Nevertheless, transcription of the canonical *DAPK1* mRNA was activated only upon antibody–drug combination treatment, but not after inhibiting HDACs, which only activates transcription from cryptic TSSs, located within *DAPK1* intron 2 and gives rise to novel isoforms of the gene. These results prompted the authors to develop a genome-wide analysis of the effects of inhibiting DNMTs and HDACs on the generation of alternative TSSs. They found that after these inhibitory treatments, thousands of alternative TSSs began to be used in association with the appearance of H2AK9ac, H3K14ac and H3K23ac marks in the cryptic promoters [77]. Obviously, these circumstances have to be considered when using inhibitors of DNA and histone modifications as epigenetic drugs (see Section 7).

Guo et al., working with human papillomavirus-related oropharyngeal squamous cell carcinoma, identified, by RNA-seq, 109 alternative mRNA sequences which are unique to these type of tumors [78]. The genome location of AS events correlated with high-levels of H3K27ac mark. To check the functional relation of this mark with AS, two human papillomavirus-related head and neck squamous cell carcinoma cell lines were treated with an inhibitor of BRD4, a reader of H3K27ac. This treatment reduced the cancer-specific AS and inhibited the growth of cancer cells [79]. These results are important, as they manifest the role of epigenetically-driven AS in cancer progression.

The acetylation of H3K27 also indirectly participates in a complex example of tumor-related AS, involving the inclusion of a cryptic intron. The human *AR* gene, coding for the androgen receptor, contains 8 exons and its mRNA is spliced to give several variants. The AR-V7 isoform includes a cryptic exon, located in the intronic sequence between exons 3 and 4. Its expression is 20-fold higher in hormone-refractory prostate cancer than in hormone-naïve prostate cancer [80] and it has been associated with poor patients’ prognosis [81]. Knocking down the histone demethylase Jumonji domain containing (JMJD) 1A, an eraser of H3K9 methylation, reduced the level of AR-V7 in prostate cancer cells. A complex of the splicing factor hnRPNF with JMJD1A binds the regions of the cryptic exon containing methylated H3K9 and favors the inclusion of this exon [82]. It has been recently described that, while dimers of the canonical AR bind an AR-responsive element at the promoters of suppressor genes and activates their transcription, heterodimers formed by the canonical AR and AR-V7 repress the expression of suppressors by recruiting NCOR, which in turn tethers HDAC3, resulting in the elimination of the activating mark H3K27ac. This chain of events seems to facilitate the development of castration-resistant prostate cancer [83], but a recent finding further complicates the matter. Another related enzyme, JMJD6, has been shown to recruit the splicing factor U2AF65 to the specific splicing sites to yield AR-V7 in a manner depending on the catalytic activity of JMJD6 [84]. It is obvious that further research is needed to dissect the exact role of these demethylases, but, at any rate, this series of processes represent an example of the complex relationships between cancer, splicing and epigenetic modifications.

More classical cancer-related AS events linked to histone PTMs have also been described in recent years and exon skipping is the most frequent one. The aberrant splicing of *MLH1*, a mismatch repair gene, has been studied in a cohort of 30 gastric cancer patients. The skipping of exons 10, 10–11, 16 and 17 was much more abundant in tumor tissues than in paired normal samples, as well as in gastric cancer cell lines when compared with cell lines derived from normal mucosa. Most of these aberrant AS events lead to non-functional transcripts, so the mismatch repair is impaired in patients displaying those AS patterns. ChIP analyses revealed that the level of H4K16ac and H3ac is lower in exons 10–11 of gastric cancer cell lines than in normal mucosa cells and that H3K36me3 and H3K4me2 levels were lower in exons 10–11 and 16–17 regions in cancer cells when compared with normal ones [85]. A combination of in silico and experimental approaches, integrating RNA-seq and H3K79me2 ChIP-seq data, revealed that a significant correlation exists between the epigenetic modification of H3 and two AS events, exon skipping and alternative 3′ site usage. This H3K79me2-related AS seems to drive acute myeloid leukaemia, as deletion of DOT1L1, which is the sole writer responsible for H3K79 methylation, results in both exon skipping and proliferation of leukaemic cells [86].

Following a different approach, a novel, short *ATG12* variant has been recently found in clear cell renal cell carcinoma [87]. Exons 2 and 3 are skipped in this variant and a cryptic exon located at the intron between them is included. The appearance of the short variant depends upon the reduction in the level of H3K36me3, caused by downregulation of the histone methyltransferase SETD2. The short *ATG12* variant is formed at the expense of the canonical isoform and, as it lacks the functionality of the latter in autophagy [87], this may be one of the causes by which inactivating mutations of SETD2 are associated with the development of clear cell renal cell carcinoma. Actually, the AS of *ATG12* is just one example of the effects of inactivation of SETD2, as it has been found that the loss of function of this enzyme is linked with many aberrant AS events, including intron retention in several types of cancer [88,89]. Nevertheless, H3K36me3 is not associated with intron retention in chronic myeloid leukaemia [69].

The correlation of the H3K36me3 mark with skipping also depends on histone acetylation [90], but the exact mechanisms linking these epigenetic modifications and splicing are not fully understood. In some instances, the participation of the splicing factor SRSF2, which binds exonic splicing enhancers and facilitates the formation of the spliceosome is required [85], but is not necessary in others [90]. At any rate, correlation of skipping and the presence of H3K36me3 and their influence in cancer has been demonstrated in many genes, but the mechanisms involved are complex and may require the participation of ncRNAs [91]. This issue will be discussed in the next section.

The unusual H3R2 methylation has been reported as a novel player in AS regulation. UHFR1 is a multifunctional protein, which is known as a reader of DNA and histone methylation [92,93]. More recently, it was found that it recognizes unmodified H3R2 through its PHD finger domain [94] and the role of UHFR1 and H3R2 methylation is based on this property. UHRF1 interacts with various splicing factors, but these factors are only tethered to the chromatin when H3R2 in the target nucleosomes is unmethylated [95]. Knocking down of UHRF1, which is upregulated in several types of cancer, induces changes in the AS events of two cancer cell lines [95].

A somewhat different case is that of *KRAS* gene, which is often mutated in tumors [96] resulting in the accumulation of the active GTP-bound KRAS, and, therefore, in constitutive activation of downstream signalling pathway. Human *KRAS* is transcribed to four mRNA isoforms, although only two of them, termed KRAS-4A and KRAS-4B, are translated to proteins and differ in their oncogenicity. By using Nuc-ChIP assays, which allow the quantitative detection of histone PTMs at the single nucleosome level [9], we have studied the epigenetic determinants involved in the selection of these isoforms [97]. The results obtained with colorectal cancer cell lines revealed significant differences in H3K4me3, H3K27me3, H3K36me3, H3K9ac, H3K27ac and H4K20me1 in the nucleosomes positioned around the splicing sites. The inhibition of histone-modifying enzymes alters the ratio 4A/4B, indicating that a causal relationship exists between these epigenetic modifications and the isoform selection during splicing.

Histone modifications at the promoters may also influence mRNA splicing because the transcriptional machinery may recruit several splicing factors before leaving the promoter; these factors will travel with RNA polII during transcriptional elongation [47]. The presence of these factors in the promoter depends, in turn, on the chromatin organization, which includes the epigenetic marks on nucleosomes around the TSS. A well-documented example of this type of splicing regulation in cancer has been provided by the elegant work of Zheng et al. [98] on the *FASTK* gene, which suppresses Fas-mediated apoptosis. PHF5A, a component of U2 snRNPs, is frequently upregulated in breast cancer and its knocking down induces the switching of canonical *FASTK* mRNA to an inactive, intron-retained variant. PHF5A acts in combination with SF3b, which is required for spliceosome stability. PHF5A contains a PHD-like domain, which functions as a reader of H3K4me3 and binds nucleosomes containing this mark, but not those with unmethylated H3K4. All these data allowed the authors to conclude that in cancer cells, the complex PHF5A–SF3b binds the H3K4me3-containing promoters and then is recruited by the transcriptional machinery facilitating the AS of *FASTK* to yield the inactive variant. In this way, apoptosis is suppressed allowing cancer cells to proliferate. On the contrary, in normal cells, as the PHF5A level is low, the assembly of SF3b to the *FASTK* promoter does not occur, transcription of gene results in the formation of the pro-apoptotic canonical FASTK [98]. PHF5A is also involved in an acetylation-related AS disturbance in cancer cells by a different and complex mechanism. This component of the splicing machinery can be acetylated by p300 and acetylated PHF5A reduces intron retention in KDM3A mRNA in CRC cells. As a result, this mRNA is stabilized, the histone demethylase level increased and the Wnt pathway activated, with the concomitant augmentation of cell proliferation [99].

### 2.3. Binding of ncRNAs

Since the launching of the ENCODE project in 2003, a considerable effort has been made to characterize non-coding sequences in human DNA. Consequently, a wide variety of non-coding RNAs (ncRNAs) in a number that largely exceeds that of protein-coding genes have been identified. They regulate multiple processes and might be included among the epigenetic factors, although they usually act in an indirect manner [100]. A first mention of the influence of small ncRNAs in the control of splicing was reported in 2006 [101]. Since then, regulatory roles in AS have been described for long ncRNAs (lncRNAs), micro RNAs (miRNAs), small nuclear RNAs (snRNAs), small nucleolar RNAs (snoRNAs) and circRNAs (see, for instance, [102]). The two first species are the most important modifiers of AS and they will be especially dealt with in the present review. Due to the huge number of data reported in the literature, with some small exception, only the reports published in the last three years will be reviewed here.

MiRNAs are short (21–24 nucleotides), non-coding RNAs. The mature miRNAs may bind through base pairing the 3′UTR of target mRNAs and downregulate their expression, especially by inducing their degradation or by inhibiting translation. Their main role in AS is an indirect one: by targeting the mRNA of some splicing-related factors, they eventually cause shifts in AS patterns of several genes as will be considered in Section 2.5.

A remarkable example is that of the action of miR-574-5p on *PTGES*, the gene coding for the microsomal prostaglandin E synthase 1 (mPGES-1). This miRNA enhances the stability of *PTGES* mRNA favoring tumor growth and additionally induces the formation of a novel *mPGES-1* isoform, which includes part of the 3′ UTR, from which its middle region is spliced out [103,104]. This process apart from representing a novel mode of action of miRNAs, involves a novel type of AS. The mechanistic reasons for the latter are not yet well understood.

The second group of ncRNAs considered here is that formed by lncRNAs, which are more than 200 nt long. Their role in AS has been recently reviewed [105] and only some recent findings will be commented here.

Prostate cancer-associated transcript 6 (PCAT6) is a lncRNA that sponges miR-326, an inhibitor of hnRNPA2B1 expression. PCAT6 is overexpressed in hepatocellular carcinoma and, consequently, the miR-326 level is reduced and that of hnRNPA2B1 enhanced [106], but the exact AS events effected have not yet been determined.

Metastasis-associated lung adenocarcinoma transcript 1 (MALAT1), with a length of around 8000 nucleotides, is perhaps the most important lncRNA associated with AS. It was early reported that MALAT1 may sequester SR proteins to nuclear speckles and therefore, buffers the presence of these splicing factors in the nucleoplasm. Actually, its downregulation increases the levels of active SR proteins in the nucleoplasm, resulting in an enhanced exon inclusion in many genes [107]. Recently, it has been described that the expression of MALAT1 is downregulated after active exercise as a consequence of the pro-oxidant cellular environment [108]. These results emphasize the importance of a healthy lifestyle in the prevention of cancer-related deleterious AS events.

A less common class of ncRNAs is that consisting in tRNA halves from the 3′ or 5′ termini, which are usually known as tiRNAs. A 33 nt from the 5′ terminus of tRNA^Gly^, further referred to as tiRNA-Gly, which has been recently reported to be increased in papillary thyroid cancer and to alter AS. Although several mechanisms are implicated in its mode of action, for the purpose of the present review it may suffice to mention that tiRNA-Gly induces several AS events among which is the exon 16 splicing in *MAP4K4* pre-mRNA. This effect, which is mediated by the tiRNA-Gly-induced stabilization of the spliceosome component RBM17, enhances proliferation and migration of cancer cells involving the MAPK pathway [109].

### 2.4. Remodeling of Chromatin

Remodeling of chromatin is often considered as an epigenetic factor because it may alter gene expression without modifying DNA sequence. Changes in nucleosome positioning and/or stability may allow some factors to bind their target sequences. While this often results in alterations in gene expression, it may also give rise to changes in AS. These are achieved by changes in the binding of splicing factors or by the direct interaction of members of the remodeling machines with spliceosome components [110].

The role of the three SWI/SNF mammalian remodeling complexes in cancer has been widely studied. They differ in some complex-specific subunits, but the core ATPase catalytic subunit is, in all the complexes, one of the two mutually exclusive BRG1 or BRM enzymes, coded, respectively, by the genes *SMARCA4* and *SMARCA2* [16]. Curiously enough, the BRM-containing complexes are involved in AS as a result of the interaction of the core ATPase with spliceosome components [111], while BRG1 does not influence AS [112]. The interaction of BRM with the splicing machinery leads to the inclusion of variant exons in the mature mRNA of several cancer-related genes [113], but it remains to be determined whether these AS events are crucial for tumor development. The role of BRM-driven AS in cancer is further complicated by the fact that the *SMARCA2* gene may be transcribed to 9 mRNA isoforms, which give rise to five different proteins [114]. Another example in which a component of the remodeler is spliced is provided by cBAF, one of the members of the human SWI/SNF complexes. It is characterized by the presence of one of the two mutually exclusive AT-rich interaction domain (ARID)-containing proteins, ARID1A or ARID1B. The Ewing’s sarcoma oncoprotein EWS/FLI1 alters the splicing of *ARID1A* mRNA, leading to the preferential selection of the long isoform ARID1A-L, responsible for sarcoma growth [115]. A last example of the involvement of chromatin remodelers in cancer-associated AS is provided by MORC2, a DNA-dependent ATPase first described as involved in remodeling of chromatin during response to DNA damage [116]. The mutation M276I, reported in some triple-negative breast cancer patients, enhances its binding to hnRNPM, with the result of switching the splicing of CD44 from its normal isoform to the mesenchymal one [117].

A different type of aberrant AS related to alterations in chromatin structure has been recently described to occur in a broad variety of cancers. It is caused by the upregulation of short H2A variants, especially of the H2A.B type, which are normally expressed only in testes. The aberrant incorporation of H2A.B in chromatin results in nucleosome destabilization, and a recent analysis of TCGA database has revealed the appearance of thousands of altered splicing events in cancers [118]. Although the mechanisms are not known, the kinetic coupling effects might account for the relationship between H2A.B destabilization of nucleosomes and altered AS patterns.

### 2.5. Epigenetic Regulations of the Splicing Machinery

**Figure 3:**
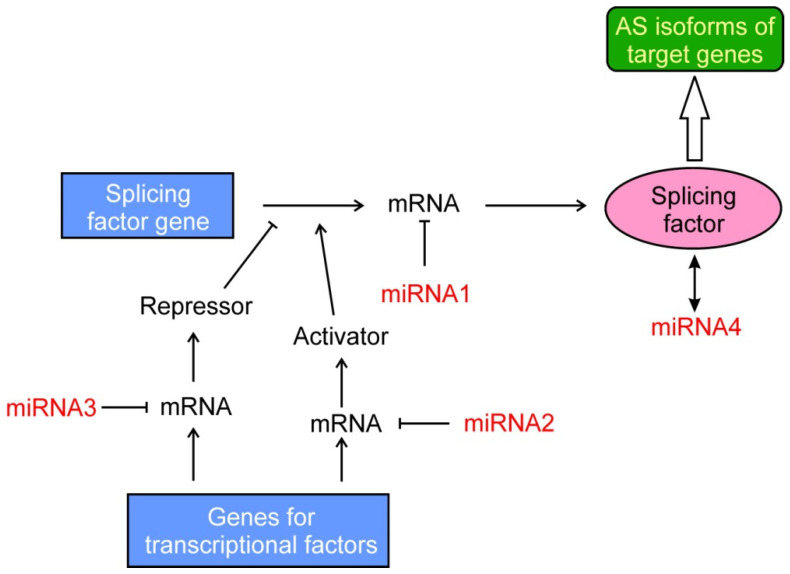
Ways in which miRNAs may ultimately affect alternative splicing. First, miRNAs (exemplified by miRNA1) may target the mRNA of a splicing factor, resulting in the reduction of its level. A second possibility is that the function of miRNAs, such as miRNA2 or miRNA3, results in a change of the levels of activators or repressors of the transcription of the splicing factor gene. Finally, some miRNAs (as miRNA4) may interact directly with the splicing factors.

As mentioned above, miRNAs often target mRNAs of splicing factors, with the subsequent inhibition of their translation (Figure 3). This mechanism was first reported to occur in 2007 [119,120] and in the last years several examples have been described. It must be noted that the downregulation of a single splicing factor may result in many shifts in the splicing patterns. For instance, the inhibition of hnRNPF by miR-139-5p results in the alteration of 174 splicing events [121]. The case of the related miR-193a-5p deserves special attention, because targeting the splicing factor SRSF6 alters the splicing of *OGDHL* and *ECM1* genes. Curiously enough, inhibition of SRSF6 in the first gene results in the inclusion of an exon, while in the second gene an exon skipping takes place. Nevertheless, the two aberrant resulting isoforms coincide in that they both activate EMT in pancreatic cancer [122].

Apart from DNA methylation (Section 2.1), miRNAs also play a role in the shift from the normal pyruvate kinase PKM1 to isoform PKM2, responsible for the Warburg effect in tumors. This issue has been recently covered by the exhaustive review of Taniguchi et al. [123] and only the papers published afterwards are mentioned here. It has been demonstrated that overexpression of miR-206, downregulated in cancer cells, induces the shift from PKM2 to PKM1, reducing the Warburg effect and that this miRNA targets the splicing factor hnRNPA1 [124]. A novel miRNA, miR-339-5p, also has been found to target hnRNPA1 as well as PTBP1 in colon cancer cells to reduce the PKM2/PKM1 ratio [125].

A particular example of miRNA-mediated silencing of splicing factors, which includes a further complication, is provided by miR-200c and miR-375. The *QKI* gene encodes an RNA-binding protein, which regulates pre-mRNA splicing, export of mRNAs and mRNA stability. However, the *QKI* mRNA itself is subject to splicing, giving rise to three major isoforms. It has been found that miR-200c and miR-375 bind the 3′-UTR of the isoform *QKI-5*, which considerably differs from those of the other two isoforms. QKI-5 affects the splicing of hundreds of genes during EMT and hence, its suppression has considerable influence on the spreading of cancer cells [126]. A recent example is that of the *ADD3* gene which codes for a membrane protein involved in cell adhesion and migration. In normal lung tissues, exon 14 is skipped, as the binding of QKI-5 inhibits its splicing, but the downregulation of that factor in lung cancer results in the inclusion of exon 14 to give an isoform that promotes proliferation and migration of cancer cells [127]. The mechanism of action of QKI-5 is a singular example of how miRNAs may influence AS through suppressing a spliced isoform of a splicing factor. Other reports dealing with the cancer-specific targeting of splicing factors by miRNAs have been published [128,129], but the final AS events produced are not yet known.

In some occasions, miRNAs affect splicing in a double indirect manner, namely, by targeting the mRNA of a transcriptional factor that, in turn, activates or represses a splicing factor (Figure 3). For instance, miR-133a targets the *GRHL2* mRNA and the downregulation of this transcriptional factor reduces the level of the splicing factor ESRP1, which finally results in the isoform switching of adherens junction-associated protein p120-catenin, with the eventual loss of E-cadherin and EMT induction in airway epithelial cells. Interestingly, this whole chain of events has been proved to occur in healthy mice exposed to cigarette smoke [130]. In this context, it has been recently found that the cirRNA circ-0005585 sponges miR-23a/b and miR-15a/15b/16, which trigger the downregulation of ESRP1. Therefore, the action of circ-0005585 finally results in the transition from the mesenchymal to epithelial phenotypes in ovarian cancer cells [131]. An indirect mechanism is also involved in the role of miR-21 on the abnormal AS found in several patients with myelodysplastic syndrome. The micro RNA targets *SKI* mRNA, with the concomitant lowering of hnRNPK activity and alteration in AS [132].

A different example of epigenetic modification of splicing factors has been recently described by the Esteller’s group. CELF2 is an RNA binding protein that targets at (CUG)_8_ and UG repeats and at UGUU motifs, and is involved in the regulation of splicing (reviewed in [133]). The *CELF2* gene is silenced in human breast cancer by promoter hypermethylation, and the restoration of its expression reduces cell proliferation; this allowed the authors to propose that *CELF2* behaves as a tumor suppressor. After an RNAseq analysis they found that *CELF2* expression affects the AS patterns of several genes, some of them coding for key players in cancer progression, such as the autophagy protein ULK1 and the apoptotic factor CARD10 [134].

## 3. Alternative Splicing of Epigenetic Writers, Erasers or Readers

The epigenetic modifications that result in altered AS have been considered until now. In a certain sense, present section covers an opposite relation between AS and epigenetics, namely, the cases in which aberrant AS of epigenetic factors–writers, erasers or readers–ultimately results in cancer development.

Concerning the writers, the full-length histone methyltransferase EZH2, which catalyzes the introduction of the repressive mark H3K27me3, may favor EMT by inhibiting some suppressor genes, such as *EFNA5*, through a mechanism involving the recruitment of the enzyme by the zinc-finger factor ZNF518B [135]. The *EZH2* gene possesses 20 exons; the inclusion of the alternative exon 14, driven by the overexpression of the upregulation of the splicing factor SF3B3, results in the increased proliferation of clear cell renal cell carcinoma. Contrarily, a variant lacking exon 14, which is necessary to build up the active site, acts as a transcriptional repressor of genes involved in cell cycle progression, and its overexpression inhibits cell growth and migration [136]. This variant was subsequently identified as a developmental factor of the central nervous system [34]. A variant of the protein arginine methyltransferase PRMT1, named PRMT1Δarm, lacks the exons required to organize the dimerization domain required to achieve enzymatic activity. Consequently, PRMT1Δarm is unable to methylate arginines, but it retains the chromatin-binding capacity competitively inhibiting the binding of active PRMT1 and eventually leading to increased malignancy [33].

In a similar way, aberrant AS of erasers may also lead to carcinogenesis. For instance, the BHC80-2 splice variant of the component of histone demethylase complexes BHC80 is highly expressed in treatment-induced neuroendocrine prostate cancer. BHC80-2 localizes to cytoplasm, where it triggers the pathway that increases RNA stability of multiple tumor-promoting cytokines [36].

Some splicing variants of readers have also proved to possess oncogenic properties. The differential role of the two isoforms of the methyl CpG binding domain protein 2 (MBD2) in oncogenesis is known [38,39], although there are some conflicting data. A mechanistic insight into this question has been recently published [137]. Hypoxic conditions favor the selection of the long isoform MBD2a, by repressing, via the action of miR222, the SRSF-mediated AS leading to MBD2c. Both isoforms possess the methyl-CpG binding capacity, but only MBD2a has a domain capable of recruiting the nucleosome remodeling and deacetylase (NURD) complex, which represses some genes coding for tumor suppressors.

## 4. Other Alternative Splicing Events Causing Cancer

The aberrant AS of the epigenetic writers, erasers or readers may result in cancer by altering the epigenetic state of genes involved in cancer, as commented in the preceding section. However, a different situation has been described in which a splicing isoform of a protein non-directly related to epigenetic modifications, finally results in them. The *CDH1* gene encodes E-cadherin and its transcription is epigenetically controlled by the modification of the promoter histones. The mechanism by which Snail induces metastasis is that represses *CDH1* transcription by recruiting the histone deacetylases HDAC1 and HDAC2 and the methyltransferase EZH2. The complex HDAC1/HDAC3/Snail/EZH2 is stabilized by CPEΔN, a truncated isoform of the carboxypeptidase E and, in this way, aberrant AS of a gene normally unrelated to epigenetic processes, results in the induction of metastasis in lung cancer through the epigenetic inhibition of a suppressor of invasion [138].

A somewhat related mechanism was described by Chang et al. [139]. The transcripts of the *SSP1* gene, coding for the extracellular matrix protein osteopontin, contain three major variants, named *OPNa*, *OPNb* and *OPNc* [140]. *OPNc* level increases in CRC cells after treatment with 5-fluorouracil and this isoform may be related to chemoresistance to conventional chemotherapy. As commented above, skipping of exon 4 that yields *OPNc* is facilitated by HDAC1 or HDAC2 and by the transcriptional factor RUNX2 [71], but they are also controlled by the methyl-CpG binding protein MeCP2 when phosphorylated at S421, a modification possibly regulated by calmodulin-dependent protein kinase II [139]. Therefore, in this case the epigenetic modification of an epigenetic reader controls an AS event leading to chemoresistance.

The role of lncRNAs in cancer-related AS has been commented in Section 2.3. Now, it has been mentioned that lncRNAs also experience AS. A short splice variant of lncRNA RP11-369C8.1, known as TRMP-S, promotes progression of cancer cells through cell cycle by reducing the expression of the suppressor p27. This effect results from several mechanisms, among which the stabilization of UHRF1 is relevant to this review. Shuai et al. [141] have found that TRMP-S facilitates the interaction of UHRF1 with the deubiquitinating enzyme USP7, which prevents the proteasomal degradation of UHRF1. It has been previously mentioned that the level of this multifunctional protein is elevated in several cancers and that it interacts with various splicing factors, so TRMP-S may also affect AS in cancer.

## 5. The Components of the Splicing Machinery: Active and Passive Roles in AS-Related Cancer Development

So far, splicing factors and spliceosome components have been considered as players of their obvious role, namely, directing AS of pre-mRNAs. That is what we call “active role”. Nevertheless, they can also play a “passive role”; in other words, their pre-mRNAs may also be spliced, generating distinct isoforms and some of these isoforms may possess oncogenic properties.

A first indirect example is provided by the *CEACAM1* gene, which encodes a protein adhesion molecule often silenced in cancer. Both the interferon response factor 1 (IRF1) and a variant of hnRNPL (Lv1) are responsible for *CEACAM1* silencing by inducing a chromatin remodeling process mediated by changes in H3K36me3 marks, which impede IRF1 access to the gene promoter [142].

AS of the gene *HNRNPA2B1* produces two main mature hnRNP isoforms, hnRNPB1 and hnRNPA2. The former isoform is anti-apoptotic and it was proposed as a marker for early detection of lung cancer [143]. The oncogenic mechanism of hnRNPB1 consists in that this isoform switches the *BCL2L1* pre-mRNA splicing from the short isoform to the long one of Bcl2, which inactivates pro-caspase 3 cleavage [144]. A more complex mechanism is involved in the splicing of *hnRNPH1* in mantle cell lymphoma. Intronic mutations surrounding exon 4 result in a distinct imbalance of *hnRNPH1* isoforms and are associated with reduced overall survival of patients. Although the molecular outcomes are not fully understood, it is clear that these intronic mutations affect the pre-mRNA binding sites of hnRNPH1 [145].

## 6. Diagnostic and Prognostic Value of Splicing-Related Epigenetic Marks

Knowledge of RNA splicing in cancer may lead to discover novel biomarkers useful for cancer diagnosis, prognosis and therapy [146]. While the last subject will be dealt with in the next section, the present one summarizes the recent findings in the first two topics, although, due to the nature of the present review, only those involved in some manner with epigenetics and related to cancer diagnosis and/or prognosis will be considered. Table 1 summarizes the recent findings in this field. Most of the epigenetic players or AS events recorded in the table has been proposed as potential prognostic markers, but some of them may prove to be valuable aids in cancer stratification.

## 7. Therapeutic Possibilities

As mentioned above, epimutations, in contrast to genetic mutations, are reversible. In this way, many of the epigenetic-driven changes in AS that cause cancer may be reversed, at least potentially. Actually, several epigenetic drugs have been developed, some of them have been approved for clinical use and others are under clinical trials (Table S4). In a similar way, drugs targeting the AS mechanisms are also clinically available or under clinical trials (Table S5) and several other potential drugs are under pre-clinical research. Apart from these possibilities, there are also other druggable targets indirectly related to the subject of the present review, which are summarized in Table 2. In some instances, the possible treatments involve the use of approved drugs for cancer or of others licensed for the treatment of a non-cancerous disease.

Several possibilities of targeting miRNAs involved in AS-related cancer have been proposed. When miRNAs play a tumor suppressor role, the use of miRNA mimics, which behave similarly to endogenous miRNAs, may be potentially used in clinics. For oncogenic miRNAs, lowering their level with anti-miRs may be the method of choice. Both procedures have been assayed in preclinical studies with promising results (reviewed in [152]). Obviously, in all the above cases, finding an efficient method to deliver miRNAs or their related molecules is an appealing challenge. Nanotechnology-based gene delivery is a promising method to reach that goal [153].

Arginine methylation, either asymmetric, catalyzed by type I PRMTs, or symmetric, catalyzed by type II PRMTs, affects pre-RNA splicing, alternative polyadenylation and transcription termination [148], and the influence of this PRMT inhibition on AS-related dependence of tumor growth is also shown in Table 2. However, the substrates of all these enzymes include, apart from histones, non-histone proteins, among which several components of the splicing machinery are included. Therefore, it is not known whether the suppression of aberrant AS is due to true epigenetic events. A similar situation occurs when using drugs targeting histone acetylation, because many HATs may also acetylate components of the splicing machinery, which are differentially acetylated in cancer cells [99,154].

## 8. Future Prospects

Apart from actual epigenetic mechanisms, i.e., those directly affecting the genetic material components, post-translational modifications of splicing machinery have been extensively reported. All these procedures offer a promising way of treating, at least, some types of cancer. Nevertheless, although there are many epidrugs available and some procedures to correct aberrant splicing are being assayed, to the best of our knowledge, the pharmacological treatment of cancer-related, epigenetic alterations of splicing has not been attempted to date. A first possibility would be to start preclinical assays of the drugs reported in Table 2, but also the drugs mentioned in Tables S4 and S5 might be assayed to check whether they are able to break the triangle cancer-AS-epigenetic modifications in some of the many malignancies mentioned in this review.

To achieve this goal, a deeper knowledge of the mechanisms of AS and of its epigenetic regulation is required, not to speak of the possible side effects that the inhibitors mentioned in Section 7 may display. Anyway, all these efforts will open a door of hope in managing cancer.

A limitation of the studies mentioned here is that in most cases, research has been carried out with tumor tissues, in which a large-cell heterogeneity exists. It can be expected that in the near future, epigenetics of AS may be studied at the single-cell level. In this manner, a more precise view of the problem will be achieved. The use of organoids derived from cancer tissues may also provide a novel way to study the functional consequences of the use of both epigenetic and AS-related drugs in cancer development.

## 9. Conclusions

The title of this review suggests that the triangle formed by the influence of AS and epigenetic changes on cancer is not necessarily a dangerous one. As mentioned under Section 7, several therapeutic strategies have been proposed, based both on the reversibility of epigenetic changes and on the targeting of AS-related processes. The therapeutic approaches mentioned are those explicitly suggested in the last years for cancers in which an epigenetics-related AS occurs. A larger number of malignancies involving this kind of relation between epigenetic alterations and aberrant AS have been referred to in this review, and it would be worth checking the possibility of pharmacologically interfering with the pertinent mechanisms as a method of cancer therapy.

## Figures and Tables

**Table 1:** Epigenetics-related AS events proposed for cancer prognosis or classification.

Epigenetic Partner	AS Event	Type of Cancer	Potential Role	Go to Section	Ref.
JARID	RBP2-H1 splice variant	MM	prognostic marker	3	[35]
miR-574-5p	PGES-1 3′UTR isoform	NSCLC	stratification marker	2.3	[103]
TET3, DNMT3A/B	ESRP1	BC	prognostic marker	2.1	[59]
miRNA-133b	miRNA targets SF3B4	HCC	prognostic marker	2.5	[129]
PCAT	downregulation of hnRNPA2B1	HCC	prognostic marker	2.3	[106]
miR-30c	miRNA targets SF2	PCa	prognostic marker	2.5	[128]
JMJD6	AS of *AR* giving AR-V7	CRPC	prognostic marker	2.2	[84]
*miR-193a-5p*	miRNA targets SRSF6	PCa	prognostic marker	2.5	[122]
miR-23a/b miR-15a/15b/16	ESRP1 expression controlled by the miRNAs	EOC	prognostic marker	2.5	[131]
miR-139-5p	miRNA targets hnRNPF	THCA	prognostic marker	2.5	[121]
KDM3A	PHF5Aac increases level of KDM3A	CRC	prognostic markers	2.2	[99]
DNAme	*BORIS* hypomethylation	HCC	prognostic marker	2.1	[64]
DNAme	exon methylation	LC	prognostic marker	2.1	[60]
PHF5A	*FASTK* intron retention	BC	prognostic marker	2.2	[98]
DNAme	*CELF2me* aberrant AS	BC	prognostic marker	2.5	[134]
DNAme	*FGFR2-IIIc*	GC	prognostic marker	2.1	[58]
SETD2	short *ATG12* at the expense of canonical *ATG12*	RCC	prognostic marker	2.2	[87]
HDAC1/HDAC3/ EZH2	increased CPEΔN	LC	prognostic marker	4	[138]
*CDKN2B-AS1* *UBE2SP1*	related to AS according to informatics analysis	HCC	prognostic markers	2.3	[147]
SETD2	involved in AS of several genes	CRC	predictive of TNM status	2.2	[89]

The epigenetic partners (writer, reader or eraser) or the AS events which might serve as a prognostic or stratification marker are typed in bold characters. The “Go to section” column indicates the main text section in which the full account on the question is given. Abbreviations for cancer types: BC, breast cancer; CRC, colorectal cancer; CRPC, castration-resistant prostate cancer; EOC, epithelial ovarian cancer; GC, gastric cancer; HCC, hepatocellular carcinoma; LC, lung cancer (unspecified); MM, malignant melanoma; NSCLC, non-small cell lung cancer; PCa, pancreatic cancer; RCC, renal cell carcinoma; THCA, thyroid cancer. For the abbreviations of writers, readers or erasers, see Table S1.

**Table 2:** Possible therapeutic targets related to alternative splicing aberrations in cancer.

Therapeutic Target	AS Event Involved	Cancer Characteristics	Molecular Mechanisms	Possible Drug	Refs.
miR-193a-5p	2 aberrant isoforms which favor EMT	overexpressed in PDAC	miRNA targets SRSF6	antisense oligonucleotides	[122]
miR-206	switch PKM2 to PKM1	downregulated in CRC	miRNA targets hnRNPA1	mimic	[124]
miR-339-5p	switch PKM2 to PKM1	downregulated in CRC	miRNA targets hnRNPA1	kaempferol (activates synthesis of the miRNA)	[125]
miR-574-5p	aberrant production of microsomal prostaglandin E synthase-1 (*mPGES1*) isoform	NSCLC	miRNA is a decoy to CUG binding protein (CUGBP1), preventing its binding to 3′ UTR of *mPGES1*	antisense oligonucleotides; inhibitors of prostaglandin E_2_ synthesis	[103,104]
hypermethylated *PKM* exon 10	inclusion of exon 10 and skipping of exon 9 resulting in PKM2	squamous cell carcinoma of the buccal mucosa	(inhibits DNMT3B and reduces exon 10 methylation)	curcumin	[62]
KDM3A	acetylation of PHF5A reduces intron retention in KDM3A	upregulated in CRC	increased level of KDM3A mRNA activates Wnt pathway	KDM3Ai	[99]
PRMTs	Many changes in splicing events	PDAC, myeloid leukaemia, melanoma and other cancers	multiple proteins involved in AS are substrates of PRMTs	type I PRMTi type II PRMTi	[148,149,150,151]
hnRNPA2B1	switch hnRNPB1 to hnRNPA2	CRC and oesophageal cancer cell lines	switch Bcl-xL to Bcl-xS, caspase activation	extracts of *Cotyledon orbiculata*	[144]

Abbreviations: PDAC, pancreatic ductal adenocarcinoma; CRC, colorectal cancer; NSCLC, non-small cell lung cancer; PRMT, protein arginine methyltransferase; KDM, lysine demethylase.

## Supplementary Materials

The following are available online at https://www.mdpi.com/article/10.3390/cancers14030560/s1, Table S1: Writers, readers and erasers of DNA methylation; Table S2: Writers, readers and erasers of histone acetylation, Table S3: Writers, readers and erasers of histone methylation; Table S4: Epidrugs approved or under clinical trial; Table S5: Drugs targeting mRNA splicing, approved or under clinical trials.
