# Supplementary material for: Alternative Splicing, Epigenetic Modifications and Cancer: A Dangerous Triangle, or a Hopeful One?
Source: Cancers (Basel). 2022 Jan 22;14(3):560. doi: 10.3390/cancers14030560 (PMC8833605; doi:10.3390/cancers14030560)
Supplement: Supplementary file 1 [file cancers-14-00560-s001.zip › cancers-1527774-supplementary.pdf]

Alternative splicing, epigenetic modifications and cancer: A dangerous triangle, or a hopeful one?

Francisco Gimeno-Valiente, Gerardo López-Rodas, Josefa Castillo, Luis Franco

SUPPLEMENTARY MATERIALS

Table S1. Writers, readers and erasers of DNA methylation.

**Writers and their functions** [1]	**Readers** [2]	**Erasers and their mechanism** [3]
DNMT1	Methyl binding domain (MDB)-containing proteins	Ten-eleven translocation (TET) enzymes
Responsible for *de novo* methylation	MeCP1	TET1
	MeCP2	TET2
DNMT3A	MBD1	TET3
DNMT3B	MBD2	
Use hemimethylated DNA as substrate.	MBD3	Catalyse oxidative removal of methyl group *via* 5- hydroxymethylcytosine
Function: maintenance of methylation in mitotic division	MBD4	
DNMT3L		
DNMT3B3		
No DNA methyl transferase activity; only regulatory roles		

Table S2. Writers, readers and erasers of histone acetylation.

**Writers** [4–6]	**Readers** [7–11]	**Erasers** [12–14]
GCN5/PCAF family (GNAT)	**Bromodomain**	*CLASS I family*
GCN5 (KAT2A), PCAF (KAT2B), HAT1 (KAT1), ELP3 (KAT9), ATF2	*BRD family I*	HDAC1, HDAC2, HDAC3, HDAC8
	PCAF, GCN5L2, FALZ/BPTF, CECR2, BAZ1A	*CLASS IIa family*
MYST family	*BRD family II*	HDAC4, HDAC5, HDAC7, HDAC9a, HDAC9b
MOF/MYST1 (KAT8), MOZ/MYST3 (KAT6A), MORF/MYST4 (KAT6B), HBO1/MYST2 (KAT7), TIP60 (KAT5)	BRD2, BRD3, BRD4, BRDT	
	BRD family III	*CLASS IIb family*
	EP300, CREBBP, WDR9, PHIP, BRD8B, BAZ1B, BRWD3	HDAC6, HDAC10
p300/CBP family	*BRD family IV*	*CLASS IV family*
CBO (KAT3B), CBP (KAT3A)	BRD1, BRD7, BRD9, BRPF1A, BRPF1B, BRPF3, ATAD2, ATAD2B/KIAA1240	HDAC11
SRC family		*SIRTUINS*
SRC-1 (KAT13A), ACTR/SRC-3 (KAT13B), TIF-2 /NCOA2(KAT13C), CLOCK (KAT13D)	*BRD family V*	SIRT1-7
	TRIM66, TRIM33, TRIM24/TIF1α, SP100, SP110, SP140, SP140L, LOC93349, BAZ2A, BAZ2B	
TRANSCRIPTION CO-ACTIVATORS family		
TAF1/TAFII250 (KAT4), FIIIC220/ GTF3C1	*BRD family VI*	
	MLL, TRIM28	
	BRD family VII	
	ZMYND11, ZMYND8, TAF1, TAF1L, WDR9d1, BRWD3d1, PHIPd1	
	BRD family VIII	
	ASH1L, SMARCA2, SMARCA4, PBRM1/PB1	
	**Tandem-PHD domain**	
	MOZ (KAT6A), MORF (KAT6B), DPF1 (BAF complex), DPF2 (BAF complex), DPF3 (BAF complex)	
	**YEATS domain**	
	AF9 (YEATS3), YAF9 (YEATS4), YEATS2, ENL (YEATS1)	

Details on writers, readers and erasers can be found at http://weram.biocuckoo.org/

Table S3. Writers, readers and erasers of histone methylation.

**Writers** [11,13,15]	**Readers** [8,11,13]	**Erasers** [11,13]
**K-HMT**	**Chromodomain**	**K-HDM**
**SET domain**	CHD1, HP1, CDY1, PC1/PC2/PC/LHP1,	**FAD-dependent amine oxidase**
SUV39 family	MSL3, MRG15, CBX1-8, MPP8, Tip60,	*LSD1 family*
SUB39H1, SUV39H2, G9A, GLP, ESET, CLLL8	**PHD domain**	LSD1(KDM1A), LSD2 (KDM1B)
SET1 family	ING1-5, BPTF, RAG2, TAF3, ICBP90,	**Fe^{2+} and α-KG dependent dioxygenase**
MLL1, MLL2, SET1A, SET1B, MLL4, MLL3,	PYGO, CHD4, UHRF1 (ICBP90), DPF3,	*KDM2 family*
EZH2, EZH1	KDM5A (JARID1A), KDM5C (JARID1C),	KDM2A, KDM2B
SET2 family	KDM7D (JMJD1D), KMT2A (MLL1),	*KDM3 family*
ASH1, NSD1, NSD2, NSD3, HYPB/HIF1	KDM7B (PHF8), KDM7C (PHF2)	KDM3A-3C (JMJD1A-1C)
RIZ family	**Tudor domain**	*KDM4 family*
PRDM1/BLIMP, PDRM2/RIZ, PRDM4/PFM1	JMJD2A, 53BP1, PHF1, PHF19,	KDM4A-4D
SMYD family	PHF20, TDRD3	*KDM5 family*
SMYD1, SMYD3	**PWWP domain**	KDM5A-5D (JARID1A-1D)
SUV4-20 family	DNMT3A, BRPF1, PDP1, HDGF2, PSIP1	*KDM6 family*
SUV4-20H1, SUV4-20H2	(LEDGF)	KDM6A(UTX), KDM6B(JMJD3), KDM6C(UTY)
SET7/9 family	**MBT domain**	*KDM7 family*
SET7, SET9	PHF20L1, SFMBT, L3MBTL1/2, MBTD1	KDM7A(JMJD1D), KDM7B(PHF8), KDM7C(PHF2)
**Non-SET domain**	**WDR domain**	*KDM8 family*
DOT1L	ELP2 (STATIP1), EED, WDR5, TBL1X,	KDM8 (JMJD5)
**R-HMT**	TBL1XR1, L3MBTL2 (LIN-61)	**R-HDM**
PRMT Type-I family	**14-3-3 domain**	PAD4/PADI4, JMJD6
PRMT1, PRMT2, PRMT3, PRMT4 (CARM1),	14-3-3	
PRMT6, PRMT8	**ZF-CW domain**	
PRMT Type-II family	ZCWPW1	
PRMT5, PRMT9	**Ankyrin domain**	
PRMT Type-III family	G9A/GLP	
PRMT7		

Details on writers, readers and erasers can be found at http://weram.biocuckoo.org/

Table S4. Epidrugs approved or under clinical trial.

Inhibitors of DNA methylation		Inhibitors of HDACs		Inhibitors of bromodomains		Inhibitors of PRMTs	
**Name**	**status**	**Name**	**status**	**name**	**status**	**name**	**status**
Azacitidine	approved	Panobinostat	approved	I-BET762	CT	GSK3326595	CT
Decitabine	approved	Belinostat	approved	Birabresib	CT	AMG 193	CT
Aza-TdCyd	CT	Valproic Acid	approved	CPI-610	CT	JNJ-64619178	CT
		Romidepsin	approved	FT-1101	CT	PF-06939999	CT
		Vorinostat	approved	ZEN-3694	CT	PRT811	CT
		Pracinostat	approved as orphan drug	BMS-986158	CT	IDE397	CT
		Entinostat	CT	OTX-015	CT	PRT543	CT
		Abexinostat	CT	ABBV-075	CT		
		CUDC-101	CT	GS-5829	CT		
		Givinostat	CT	PLX-51107	CT		
		Mocetinostat	CT	TEN-010	CT		

The names of the epidrugs approved or under clinical trials (CT) are given, although some of them were approved for diseases other than cancer. Further details can be obtained from [16,17]. The progress of the clinical trials can be checked at https://www.clinicaltrials.gov/

Table S5. Drugs targeting mRNA splicing, approved or under clinical trials.

Splicing modulators		Drugs targeting enzymes involved in splicing		
**Name**	**status**	**name**	**status**	**target**
Risdiplam	approved	SM08502	CT	CLKs
Branaplam	approved as orphan drug			
Aclarubicin	CT			

The names of the drugs approved or under clinical trials (CT) are given. CLK stands for CDC2-like kinases. Further details can be obtained from [18–20]. The progress of the clinical trials can be checked at https://www.clinicaltrials.gov/

ADDITIONAL REFERENCES

1. Nishiyama, A.; Nakanishi, M. Navigating the DNA methylation landscape of cancer. *Trends Genet.* **2021**, *37*, 1012–1027.
2. Ginder, G.D.; Williams, D.C. Readers of DNA methylation, the MBD family as potential therapeutic targets. *Pharmacol. Ther.* **2018**, *184*, 98–111.
3. Wu, X.; Zhang, Y. TET-mediated active DNA demethylation: mechanism, function and beyond. *Nat. Rev. Genet.* **2017**, *18*, 517–534.
4. Roth, S.Y.; Denu, J.M.; Allis, C.D. Histone acetyltransferases. *Annu. Rev. Biochem* **2001**, 81–120.
5. Marmorstein, R.; Roth, S.Y. Histone acetyltransferases: Function, structure, and catalysis. *Curr. Opin. Genet. Dev.* **2001**, *11*, 155–161.
6. Sapountzi, V.; Côté, J. MYST-family histone acetyltransferases: Beyond chromatin. *Cell. Mol. Life Sci.* **2011**, *68*, 1147–1156.
7. Filippakopoulos, P.; Qi, J.; Picaud, S.; Shen, Y.; Smith, W.B.; Fedorov, O.; Morse, E.M.; Keates, T.; Hickman, T.T.; Felletar, I.; et al. Selective inhibition of BET bromodomains. *Nature* **2010**, *468*, 1067–1073.
8. Yun, M.; Wu, J.; Workman, J.L.; Li, B. Readers of histone modifications. *Cell Res.* **2011**, *21*, 564–578.
9. Marmorstein, R.; Zhou, M.M. Writers and readers of histone acetylation: Structure, mechanism, and inhibition. *Cold Spring Harb. Perspect. Biol.* **2014**, *6*, a018762.
10. Jain, A.K.; Barton, M.C. Bromodomain histone readers and cancer. *J. Mol. Biol.* **2017**, *429*, 2003-2010.
11. Biswas, S.; Rao, C.M. Epigenetic tools (The Writers, The Readers and The Erasers) and their implications in cancer therapy. *Eur. J. Pharmacol.* **2018**, *837*, 8–24.
12. Seto, E.; Yoshida, M. Erasers of histone acetylation: The histone deacetylase enzymes. *Cold Spring Harb. Perspect. Biol.* **2014**, *6*, a018713.
13. Hyun, K.; Jeon, J.; Park, K.; Kim, J. Writing, erasing and reading histone lysine methylations. *Exp. Mol. Med.* **2017**, *49*, e324.
14. Park, S.Y.; Kim, J.S. A short guide to histone deacetylases including recent progress on class II enzymes. *Exp. Mol. Med.* **2020**, *52*, 204–212.
15. Yang, Q.; Yang, Y.; Zhou, N.; Tang, K.; Lau, W.B.; Lau, B.; Wang, W.; Xu, L.; Yang, Z.; Huang, S.; et al. Epigenetics in ovarian cancer: Premise, properties, and perspectives. *Mol. Cancer* **2018**, *17*, 109.
16. Miranda Furtado, C.L.; Dos Santos Luciano, M.C.; Silva Santos, R. Da; Furtado, G.P.; Moraes, M.O.; Pessoa, C. Epidrugs: targeting epigenetic marks in cancer treatment. *Epigenetics* **2019**, *14*, 1164–1176.
17. Lu, Y.; Chan, Y.T.; Tan, H.Y.; Li, S.; Wang, N.; Feng, Y. Epigenetic regulation in human cancer: The potential role of epi-drug in cancer therapy. *Mol. Cancer* **2020**, *19*, 79.

18. Martinez-Montiel, N.; Rosas-Murrieta, N.H.; Ruiz, M.A.; Monjaraz-Guzman, E.; Martinez-Contreras, R. Alternative splicing as a target for cancer treatment. *Int. J. Mol. Sci.* **2018**, *19*, 545.
19. Black, A.J.; Gamarra, J.R.; Giudice, J. More than a messenger: Alternative splicing as a therapeutic target. *Biochim. Biophys. Acta - Gene Regul. Mech.* **2019**, *1862*, 194395.
20. Tang, Z.; Zhao, J.; Pearson, Z.J.; Boskovic, Z. V; Wang, J. RNA-targeting splicing modifiers: Drug development and screening assays. *Molecules* **2021**, *26*, 2263.
